# Lunatic Hospitals

**Published:** 1844-04

**Authors:** 


### LUNATIC HOSPITALS.

We take a deep interest in the reports of these benevolent institutions. Their statistics are consolatory to suffering humanity—among the proudest trophies of the healing art. We have before us, at this time, three annual reports from lunatic asylums, of which we shall give a short account. The first we shall notice is

*The Fifth Annual Report of the Ohio Lunatic Asylum*, at Columbus. Extensive additions have been made to the buildings of this asylum, by which it will be capable of accommodating, when all are completed, about three hundred and forty-five patients, which, it is supposed, will be equal to the wants of the insane population of that state. "The number of patients," says the report, "who have been inmates of the institution since the 15th of November, 1842, is 207—males 105, females 102, all citizens of the State of Ohio. The number of patients remaining in the asylum at the close of last year was 142—75 males and 67 females. During the past year 65 patients have been admitted, viz: 32 males and 33 females. The number discharged in the past year was 59, viz: 30 males and 29 females. Of these 38 were discharged recovered, 7 improved, 10 stationary, and 4 by death. Two others are convalescent, and have a strong desire to return to their friends, but we think it most prudent for them to remain a few weeks longer. As a general remark, it is much the best plan for convalescents to remain until their bodily health is firmly established, and full proof is exhibited of their entire exemption from mental disease. The few instances in which recoveries seem to have been promoted, by a return of the patient to the endearments of home and friends, are but extremely rare and uncertain exceptions to a safe and substantial general rule. By reference to our summary remarks it will be seen that, of the recent cases discharged during the past year, 100 per cent. recovered. It is very seldom that every circumstance unites to favor a result of this kind."

Since the opening of the institution three hundred and fifty-four applications for admission into it have been refused for want of room; and the report supposes, that in Ohio alone there are at this time, between three and four hundred insane persons who need the protection and care of a public hospital. Happily, it seems to be the policy of that State "to make abundant provision for the reception of every insane citizen, whether male or female, rich or poor, curable or incurable." It is a humane and wise policy, deserving of all praise.

This report abounds in statistics of great interest. We regret that we cannot copy more extensively from it, and that our limits preclude us from any notice of the seven cases related in the appendix to the report. The author of the report, William M. Awl, M.D., superintendent of the institution, is entitled to great credit for the fulness with which he has reported all the facts that interest the medical philosopher.

*The Annual Report of the Physician and Superintendent of the Eastern Asylum, at Williamsburg, Va.* This report is for 1843, and we are indebted for it to John M. Galt, M.D., superintendent and physician. "The number of patients who have been inmates of the Eastern asylum since the first of January, is one hundred and thirty-five. Of these, eighty were males and fifty-five females. Six of the male patients and nine of the females were colored persons. The number of patients on the first of January, was ninety-three, viz: males fifty-seven, females thirty-six. During the year forty-two have been received: twenty-three of these were males, and nineteen females. The number of the discharges is twelve, viz: nine males and three females; one male and one female now in the institution are convalescent. The number of the deaths is fourteen, viz: nine males and five females, or about ten and a third per cent. The number of patients at present is one hundred and nine, viz: males sixty-two, females forty-seven." From the 1st of July, 1841, to the 1st of July, 1843, or during the space of two years, 50 patients were received: of this number 24 have recovered, counting one case in the institution now convalescing. This gives a per centage of 48.51, which taken simply is very high; and under the adverse circumstances with which the institution has had to contend, must be considered still higher. We believe that there are few institutions exhibiting a higher per centage, under any thing like similar circumstances. The per centage of the Massachusetts State lunatic hospital at Worcester is 43.41: which institution, taken in all its characteristics, may be considered as the first in the world."

This report is drawn up with great care. The author feels a just and laudable pride in the management of the institution for which he is responsible, and may contemplate the fruits of his stewardship with high satisfaction. His report wears a studious and pholosophical aspect. His heart is evidently in the subject, and he has made himself master of it.

*Eleventh Annual Report of the Trustees of the State Lunatic Hospital, at Worcester, Mass.* We received a copy of this report a few days ago, and we opened it in the fullest conviction that we should find much in it to admire. "The noblest monument of the public charities of Massachusetts," as the report styles it, it is an institution which would do honor to any country in the world. "From the laying of the foundation-stone to this day," says the report, "Heaven seems to have directed the undertaking, and to have crowned with its mercies the entire work. Not to see it, we should be blind; not to acknowledge it, we should be most ungrateful." We subjoin the following summary:

"Eleven years have passed since this hospital was opened for the reception of patients; in that time there have been received one thousand and seven hundred and seventy-seven patients.

"Committed by the courts, 1311; committed by overseers of the poor and friends, 466; the number of discharges is 1522; the number of recoveries is 792; during the last year there have been in the hospital, different patients, 458; at the commencement of the year, 238; admitted during the year, 220; now remaining, 255; recovered, 116; died, 22; discharged improved, 32; discharged as harmless and incurable, 24; sent to house of correction, for want of room, by trustees, 2; discharged by the courts, as incurable and dangerous, 6; discharged by trustees' private board, incurable, for want of room, 1; average number of patients in the hospital for the year, 244 1-6.

"Of those who have recovered, ninety-four were cases of less duration than one year, and thirty-two of longer duration.

"Application has been made for one hundred and fifty-seven who were not received at the time, and forty-eight who have not been received at any time, for want of room."

A richly-merited compliment is paid by the trustees of the asylum to Dr. Woodward, the accomplished, humane, and indefatigable superintendent, whose gentleness, courtesy, skill, and energy have made the hospital, in their own language, "a model and a praise in our own and in foreign lands."

"All the American hospitals for the insane," says Dr. Woodward, "must be able to accommodate from *three* to *four thousand* patients. They must relieve a vast amount of suffering of patients and anxiety of friends. They are probably not surpassed for custodial care, or remedial treatment, by any institutions in the world."

Dr. Woodward, very justly, in our opinion, dissents entirely from the judgment of Jacobi, that the sexes should be kept in separate establishments, and be allowed no intercourse or communication with each other. He insists upon the judicious intercourse of the sexes, as being as favorable in its influence upon insane men and women, especially when convalescent, as it is upon individuals in health. He would have the insane treated as other people are, keeping out of sight as far as practicable their peculiarities, encouraging them to self-respect and rational conduct, and getting them into habits of order and accustomed channels of duty and employment. Concerning devotional exerecises, his remark is, that "our confidence in the benefits which result from religious worship for the insane increases from year to year." The experience of the other superintendents is to the same purpose. Similar declarations are made in each of the foregoing reports. Y.

